# Heterologous expression of flax *PHOSPHOLIPID:DIACYLGLYCEROL CHOLINEPHOSPHOTRANSFERASE* (*PDCT*) increases polyunsaturated fatty acid content in yeast and Arabidopsis seeds

**DOI:** 10.1186/s12896-015-0156-6

**Published:** 2015-06-30

**Authors:** Aruna D Wickramarathna, Rodrigo M P Siloto, Elzbieta Mietkiewska, Stacy D Singer, Xue Pan, Randall J Weselake

**Affiliations:** Department of Agricultural, Food and Nutritional Science, University of Alberta, Edmonton, AB T6G 2P5 Canada

**Keywords:** α-linolenic acid, *Linum usitatissimum*, Phosphatidylcholine-diacyglycerol interconversion, *AtROD1*, PUFA, *Saccharomyces cerevisiae*

## Abstract

**Background:**

Flax (*Linum usitatissimum* L.) is an agriculturally important crop with seed oil enriched in α-linolenic acid (18:3 ^*cis*Δ9, 12, 15^; ALA). This polyunsaturated fatty acid (PUFA) is the major determinant for the quality of flax seed oil in food, nutraceuticals and industrial applications. The recently identified enzyme: phosphatidylcholine diacylglycerol cholinephosphotransferase (PDCT), catalyzes the interconversion between phosphatidylcholine (PC) and diacylglycerol (DAG), and has been shown to play an important role in PUFA accumulation in *Arabidopsis thaliana* seeds.

**Methods:**

Two flax *PDCT* genes were identified using homology-based approach.

**Results:**

In this study, we describe the isolation and characterization of two *PDCT* genes from flax (*LuPDCT1* and *LuPDCT2*) with very high nucleotide sequence identity (97%) whose deduced amino acid sequences exhibited approximately 55% identity with that of *A. thaliana* PDCT (AtROD1). The genes encoded functionally active enzymes that were strongly expressed in developing embryos. Complementation studies with the *A. thaliana rod1* mutant demonstrated that the flax PDCTs were capable of restoring PUFA levels *in planta*. Furthermore, PUFA levels increased in *Saccharomyces cerevisiae* when the flax *PDCT*s were co-expressed with *FATTY ACID DESATURASES* (*FADs*)*, FAD2* and *FAD3*, while seed-specific expression of *LuPDCT1* and *LuPDCT2* in *A. thaliana* resulted in 16.4% and 19.7% increases in C18-PUFAs, respectively, with a concomitant decrease in the proportion of oleic acid (18:1^*cis*Δ9^; OA).

**Conclusions:**

The two novel *PDCT* homologs from flax are capable of increasing C18-PUFA levels substantially in metabolically engineered yeast and transgenic *A. thaliana* seeds. These flax PDCT proteins appear to play an important dual role in the determination of PUFA content by efficiently channelling monounsaturated FAs into PC for desaturation and moving the resulting PUFAs out of PC for subsequent use in TAG synthesis. These results indicate that flax PDCTs would be useful for bioengineering of oil crops to increase PUFA levels for applications in human food and nutritional supplements, animal feed and industrial bioproducts.

**Electronic supplementary material:**

The online version of this article (doi:10.1186/s12896-015-0156-6) contains supplementary material, which is available to authorized users.

## Background

Seed oils provide an important source of dietary fats in both human and livestock nutrition [1,2] and are becoming increasingly attractive in nutraceutical and bio-based industrial applications, as well as biofuel production [2,3]. The functional qualities of the various seed oils, and thus their suitability for a particular application, are primarily determined by their fatty acid (FA) content and composition. Seed oil from flax (*Linum usitatissimum* L.) is enriched in α-linolenic acid (18:3 ^*cis*Δ9, 12, 15^; ALA) [4], with conventional varieties containing 45% to 65% of this essential dietary FA. ALA is a precursor for the synthesis of very long chain omega-3 polyunsaturated fatty acids (PUFAs), which are responsible for the myriad of health benefits that have been attributed to flax oil, including positive effects with respect to cardiovascular health and inflammatory diseases, as well as anticancer properties [5-7]. As a result of this, flax seeds have been widely used in animal feed to increase the ALA content of eggs and meat [8], thus altering their FA profiles and rendering them more nutritionally attractive [7]. Furthermore, the remarkably high ALA content of flax seed oil also provides it with a superior “drying” quality, making it very suitable for widespread use in industrial and domestic products [9].

FA biosynthesis within developing seeds of oleaginous crops occurs in the plastids, with the resulting FAs being subsequently released into the cytosol predominantly as oleic acid (18:1 ^*cis*Δ9^; OA), along with minor amounts of palmitic (16:0) and stearic (18:0) acids in the form of acyl-Coenzyme A (CoA) [10,11]. OA-CoA can be further elongated on the endoplasmic reticulum (ER) or used in the acylation of *sn*-glycerol-3-phosphate. In a large number of oilseeds, the majority of OA enters the membrane lipid phosphatidylcholine (PC), where a second and third double bond can be added at the *sn*-2 position via the catalytic action of ER-localized fatty acid desaturases (FADs), FAD2 and FAD3, to produce the PUFAs linoleic acid (18:2 ^*cis*Δ9, 12^; LA) and ALA. These PUFAs, present on PC, can then be incorporated into triacylglycerol (TAG), which is the most common form of storage lipid in the seeds of many plant species and serves as a vital energy source for a number of biological functions.

The PUFAs produced in PC are known to end up in TAG via various possible metabolic routes [11]. Firstly, PUFAs can be cleaved from PC through the catalytic action of phospholipase A, with the resulting free FAs being esterified to Coenzyme A (CoA) through the catalytic action of long chain acyl-CoA synthetase. PUFAs may also enter the acyl-CoA pool through acyl-exchange with PC catalyzed by lysophosphatidylcholine acyltransferase (LPCAT). In both cases, the resulting PUFA-CoAs are then available as a source of fatty acyl chains for incorporation into TAG via the acyl-CoA-dependent *sn*-glycerol-3-phosphate pathway whereby diacylglycerol acyltransferase (DGAT) catalyzes the acylation of diacylglycerol (DAG) to form TAG. Alternatively, PUFAs can be directly transferred from the *sn-*2 position of PC onto DAG to generate TAG through the catalytic action of phospholipid:diacylglycerol acyltransferase (PDAT) in an acyl-CoA-independent manner. In the case of phospholipase A and PDAT action, the resulting lysophosphatidylcholine can be reacylated to PC via the forward reaction catalyzed by LPCAT.

A more recently discovered metabolic route for channelling PUFA into TAG involves the conversion of PUFA-enriched PC to PUFA-enriched DAG through the removal of the phosphocholine headgroup, which has been suggested to be the predominant pathway of DAG production [12,13]. This PC-derived DAG appears to be mainly produced through the catalytic action of phosphatidylcholine diacylglycerol cholinephosphotransferase (PDCT) [12,14], although smaller proportions may also be generated via the reverse action of CDP-choline: diacylglycerol cholinephosphotransferase (CPT) [15] or by the action of phospholipase C and/or D through a lipase-mediated pathway [16]. Furthermore, in a similar manner to its involvement in desaturation, PC also acts as the substrate for the production of epoxy-, conjugated-, hydroxy-, acetylenic-, and other unusual FAs [14,17-19], with acyl moieties on DAG entering PC through the action of PDCT, which are then modified and returned to DAG for further acylation catalyzed by DGAT in the *sn*-glycerol-3-phosphate pathway. Thus, while it is possible that the metabolic channelling of PUFAs and unusual FAs from PC to TAG involves more than one of the aforementioned mechanisms and may vary based on the plant species, it seems that PDCT in particular is likely to play a key role in the determination of seed oil FA composition.

In this paper, we report the isolation of two embryo-expressed *PDCT* genes from flax that share a very high level of sequence identity with one another. Functional characterization of these two genes in yeast and plant systems demonstrated that they both encode functional enzymes. Furthermore, heterologous expression of both flax genes increased PUFA levels in metabolically engineered yeast and transgenic *A. thaliana*. While mutation of a *PDCT* from Arabidopsis (*rod1*) has previously been shown to result in decreased PUFA accumulation in that species [12], this is the first instance in which the heterologous expression of a *PDCT* has been linked with enhanced production of PUFAs, providing further evidence that PDCT plays a crucial role in determining the FA profile of seed lipids. Since the use of biotechnology to improve the FA composition of seed oils to match the demand of a particular industry is fast becoming a popular approach [1,2], the flax *PDCT* genes hold great promise for the future modification of PUFA levels in a wide range of oil crops.

## Results

### Isolation of *PDCT* homologs in flax

**Figure 1:**
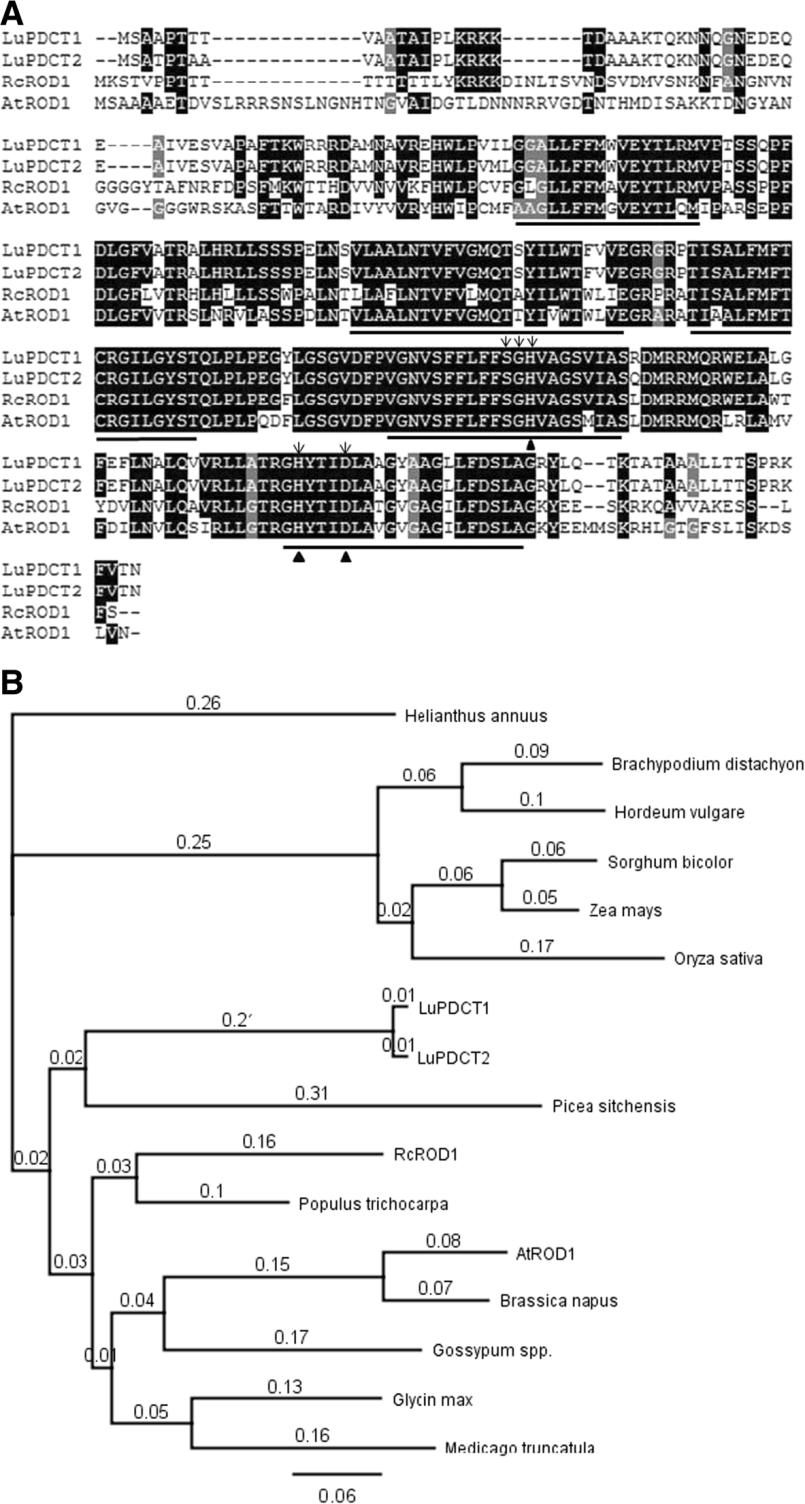
Comparison of PDCT homologs from higher plants. **(A)** Alignment of deduced amino acid sequences from LuPDCT1, LuPDCT2, RcROD1 and AtROD1. Sequences were aligned using ClustalW and shading was applied using DNA Boxshade. Identical amino acids are shaded in black, while conserved substitutions are shaded in gray. Putative transmembrane domains are underlined. The five highly conserved residues in the C2 and C3 domains of the LPT family are denoted with arrows and the catalytic triads (His, His, Asp) are indicated with triangles. Protein sequences were deduced from GenBank accession numbers: *Ricinus communis* (RcROD1), XM_002517597; and *Arabidopsis thaliana* (AtROD1), At3g15820. **(B)** Phylogenetic tree showing the relationship between deduced PDCT proteins from different plant species. The amino acid sequences were deduced from GenBank accession numbers: *Ricinus communis* (RcROD1), XM_002517597; *Arabidopsis thaliana* (AtROD1), At3g15820; *Brachypodium distachyon*, XP_003563650; *Glycine max*, XP_003528315; *Hordeum vulgare*, BAK03357; *Medicago truncatula*, XP_003604371; *Picea sitchensis*, ABK25679; *Populus trichocarpa*, XP_002327418; *Sorghum bicolor*, XP_002437259; *Zea maize*, NP_001145186; and *Oryza sativa*, NP_001058029. The *Brassica napus*, *Gossypum spp*., and *Helianthus annuus* sequences were obtained from the computational biology and functional genomic website (http://compbio.dfci.harvard.edu/compbio) with the following gene identities: *Bn*, TC171107; *Gs*, TC240631; and *Ha*, TC54879. The amino acid sequences were aligned and the phylogenetic tree was constructed using Genious v. 5.3 (Biomatters Ltd. New Zealand).

The *A. thaliana* (*At*)*ROD1* (At3g15820) sequence [12] was used to query the flax genomic sequence database (www.linum.ca) [20] using the basic local alignment search tool (BLAST) [21]. By analyzing the alignments of the positive hits, two *PDCT* homologs were identified (denoted as *LuPDCT1* and *LuPDCT2*). The full length cDNA sequences of both flax genes were cloned and subsequent sequence analysis indicated that they displayed approximately 97% identity at the nucleotide level, while the deduced amino acid sequences displayed approximately 98% identity (Figure 1A). When compared to *AtROD1*, *LuPDCT1* and *LuPDCT2* exhibited 70.1% and 71.2% identity at the nucleotide level, and 55.1% and 54.7% identity at the amino acid level, respectively. PDCT homologs have also been identified in a range of other plant species, and their deduced polypeptide sequences shared between 47.6% and 65.8% identity with LuPDCT1 and LuPDCT2 (Figure 1B; Additional file 1: Figure S1).

Topology prediction software including HMMTOP [22] and TMpred [23] identified both LuPDCT1 and LuPDCT2 as integral-membrane proteins with five transmembrane regions (Figure 1A). This finding is consistent with PDCT proteins identified previously in other plant species, such as *A. thaliana* and castor (*Ricinus communis*) ROD1 proteins, which were predicted to have six transmembrane regions [12,14]. Furthermore, PDCT belongs to a large family of lipid phosphatase/phosphotransferase (LPT) proteins [12], which contain five highly conserved residues in the C2 and C3 domains, as well as a catalytic triad (His, His, Asp). As expected, all of these conserved residues were also found in both deduced flax PDCT polypeptides (Figure 1A).

Other PDCT homologs share 47.6% to 65.8% identity with the deduced amino acid sequences of flax PDCTs (Figure 1B; Additional file 1: Figure S1). Phylogenetic analysis of the two LuPDCT proteins in relation to other higher plant PDCTs, including functionally tested enzymes from *A. thaliana* [12] and castor [14], indicated that they are more closely related to protein homologs from plants such as Sitka spruce (*Picea sitchensis*) and castor than they are to that from the model plant, *A. thaliana* (Figure 1B).

#### *LuPDCT1* and *LuPDCT2* are specifically expressed in the embryo

**Figure 2:**
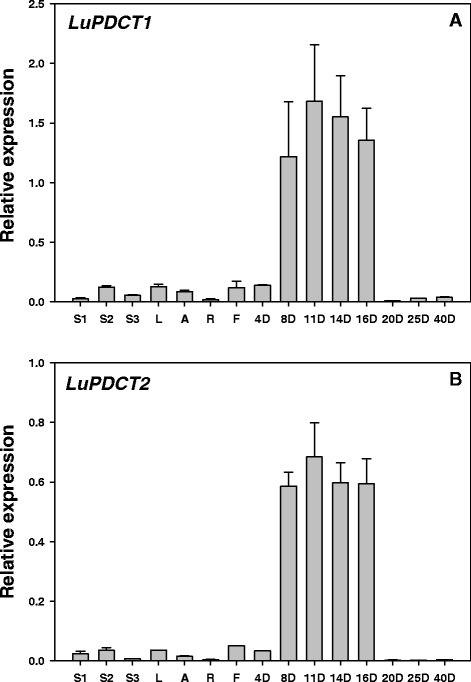
Relative expression levels of *LuPDCT1* **(A)** or *LuPDCT2* **(B)** in flax vegetative tissues, flowers, and during embryo development. Transcript levels were compared across different tissues and at different stages of embryo development using the two reference genes, *GAPDH* and *UBI2*. Data shown represent means ± SE of three biological and three technical replicates. S1, immature stem; S2, developing stem; S3, mature stem; L, leaves; A, apexes; R, roots; F, flowers; 4 to 20D, days after anthesis of developing embryos; 25D and 40D, days after anthesis of mature seeds.

To gain insight into the potential roles of *LuPDCT1* and *LuPDCT2* in flax, their expression patterns were monitored in vegetative tissues, reproductive tissues, and at various stages of embryo development using TaqMan-based qRT-PCR assays (Figure 2). The specificity of the primers used for *PDCT* genes and control gene amplification were verified by separating qRT-PCR amplicons on a 2% agarose gel. The gene-specific primers produced a single product of the desired length, confirming their specificity (data not shown).

In general, *LuPDCT1* and *LuPDCT2* exhibited similar spatiotemporal patterns of expression; however, due to differences in their primer pair efficiencies (*LuPDCT1* and *LuPDCT2* primers exhibited 88% and 97% efficiencies, respectively), it was not possible to compare levels of expression between the two genes. While both *LuPDCT1* and *LuPDCT2* were found to be expressed at only very low levels in all vegetative and floral tissues tested (stem, leaves, shoot apex, root and flowers), both genes were highly expressed during the mid- to late-stages (8 to 16 days after anthesis; DAA) of embryo development, peaking at approximately 11 DAA with transcript levels decreasing dramatically after 16 DAA to nearly undetectable levels for the remainder of seed development (Figures 2A and B). Previously, it was shown that the proportion of ALA in flax cultivar CDC Bethune embryos increased from 30% to 43% between 8 and 16 DAA, and then stabilized at approximately 20 DAA, which corresponded with a rapid accumulation of oil that increased from 1% to 20% of fresh weight between 8 and 20 DAA [24]. These results suggest that the two flax PDCT proteins may play an active role in increasing the PUFA content of the seed oil.

#### *LuPDCT1* and *LuPDCT2* encode functional PDCT enzymes

To test the enzymatic activity of the LuPDCT1 and LuPDCT2 proteins, the coding sequences of both genes were cloned into the yeast expression vector pYESBOP [24] under the control of the galactose-inducible *GAL1* promoter. The resulting vectors were transformed into the *Saccharomyces cerevisiae* mutant strain YNL 130C (*MAT*α *cpt1:: KanMX ept1*; Openbiosystems; Thermo Fisher Scientific, Huntsville, AL), which lacks CPT activity [25]. Since the PDCT activity of the enzyme encoded by the *AtROD1* gene was confirmed previously using the yeast microsomal fraction [12] and the deduced amino acid sequences of both LuPDCT1 and LuPDCT2 were predicted to have five transmembrane domains, microsomal preparations of *LuPDCT1-*, *LuPDCT2-* and empty vector-transformed cells were tested for their ability to catalyze the synthesis of PC from DAG or DAG from PC.

**Figure 3:**
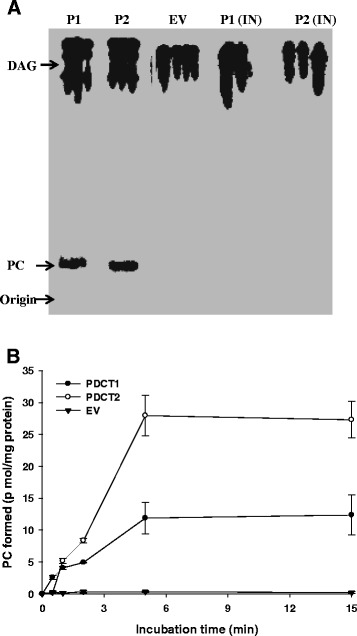
Analysis of PDCT activity of *S. cerevisiae* microsomes producing recombinant LuPDCT1 or LuPDCT2. **(A)** Production of radio-labeled phosphatidylcholine (PC) catalyzed by LuPDCT1 or LuPDCT2 following 15 min incubation. Radio-labeled substrate and product were separated using radio-thin layer chromatography (TLC) and visualized by phosphor-imaging. Microsomes from yeast YNL 130C cells transformed with pYES-LuPDCT1 (P1), pYES-LuPDCT2 (P2) or pYES (EV) were incubated with [^14^C]-glycerol- diolein in the presence of dioleoyl-PC. P1 (IN) and P2 (IN), respectively, indicate microsomes from pYES-LuPDCT1 and pYES-LuPDCT2 that were boiled prior to the assay. The phosphor image is a composite of five representative samples selected from two TLC plates which were developed under identical conditions. **(B)** Time course production of PC catalyzed by LuPDCT1, LuPDCT2 or EV microsomes. Data shown represent means ± SE based on three replicates.

For assessing the rate of formation of PC, [^14^C]-glycerol-diolein was incubated with dioleoyl-PC and yeast microsomal fraction, and the production of radiolabeled-PC was monitored after 15 min by thin layer chromatography (TLC) (Figure 3A). Radio-labeled PC was produced through the catalytic action of both LuPDCT1 (P1) and LuPDCT2 (P2), and the rate of production was found to be linear for up to 5 min (Figure 3B). In contrast, yeast microsomes bearing the empty vector control (EV) and boiled microsomes containing the recombinant enzymes, P1 (IN) and P2 (IN), failed to produce radio-labeled PC. The rate of appearance of radio-labeled PC catalyzed by microsomes containing recombinant LuPDCT1 or LuPDCT2 is shown in Figure 3B; in both cases, the enzymatic reactions were linear for up to 5 min. Microsomes containing recombinant LuPDCT1 or LuPDCT2 were also capable of catalyzing the formation of radio-labeled DAG when supplied with 1-palmitoyl-2- [^14^C]oleoyl-PC and diolein (Additional File 1: figure S2). In this latter case, time courses were not conducted and radio-labeled DAG production was only determined after 15 min of incubation. Small quantities of TAG were also produced during the reactions where production of radio-labeled DAG was monitored (Additional file 1: figure S3).

### LuPDCT1 and LuPDCT2 enhance PUFA accumulation in PC, DAG and TAG in yeast

*S. cerevisiae* has been widely used as a model organism for studying eukaryotic cellular and molecular functions [26], and yeast expression systems have commonly been used for functional characterization of plant integral membrane fatty acid desaturases such as FAD2 [27] and FAD3 [28]. However, *S. cerevisiae* does not synthesize PUFAs due to a lack of enzymes capable of introducing more than one double bond into its FAs [29], thus limiting its use in oil biosynthesis-related research involving the generation of PUFAs. To circumvent this problem, we engineered *S. cerevisiae* to produce LA and ALA by sequentially introducing expression cassettes containing cDNAs encoding LuFAD2 [30] and LuFAD3b [28].

**Figure 4:**
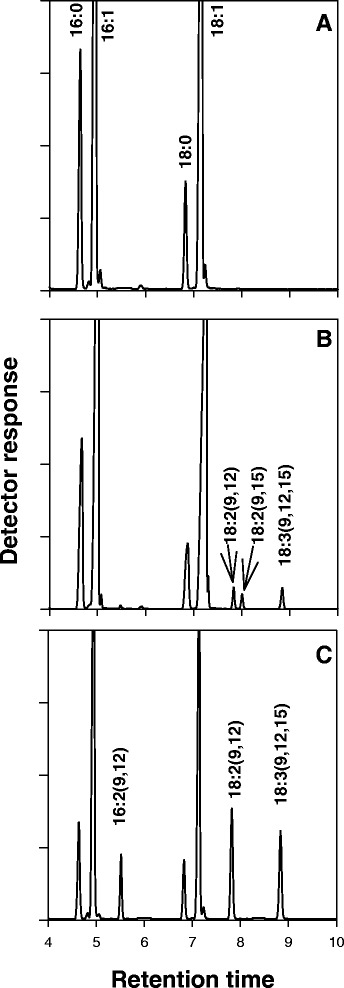
Engineering *S. cerevisiae* to produce polyunsaturated fatty acids (FAs). Gas chromatography–mass spectrometry analysis of FA methyl esters derived from the phosphatidylcholine fraction of wild-type yeast cultures transformed with the empty pYESBOP vector **(A)**, pYES + F2F3 **(B)** and pYES + F2F3 + P1 **(C)** constructs. pYES + F2F3 + P2 produced similar results to that of pYES + F2F3 + P1.

Analysis of the different lipid classes revealed that the PC fraction from wild-type (WT) yeast cells transformed with *LuFAD2* and *LuFAD3b* accumulated small but significant quantities of LA (0.64 ± 0.03%) and ALA (0.96 ± 0.15%; Figure 4, Table 1), with minute quantities of PUFAs (LA + ALA) observed in TAG and DAG fractions (Table 1). These results demonstrate that transgenic yeast cells carrying *LuFAD2* and *LuFAD3b* are capable of producing LA and ALA, particularly in PC, which are FAs not normally present in yeast. We also detected small quantities of 9, 15-octadecadienoic acid (18:2^*cis*Δ9,15^) in the PC fraction of these cells. This is consistent with previous reports [31,32] in which plant *FAD3* genes have been expressed in yeast, and is very likely due to the minor catalytic activity of higher plant FAD3 enzymes in the desaturation of monounsaturated FAs.

**Table 1:** **Fatty acid compositions of lipid classes from transgenic** ***S. cerevisae***

**Construct**	**FA composition of PC (mol %)**						
	**C16:0**	**C16:1**	**C16:2**	**C18:0**	**C18:1**	**C18:2**	**C18:3**
F2F3	5.65 ± 1.28^a^	46.55 ± 0.65	n.d.^b^	1.88 ± 0.53	43.74 ± 2.69	0.64 ± 0.03	0.96 ± 0.15
F2F3 + P1	7.98 ± 0.12	42.97 ± 0.67	4.0 ± 0.31	4.26 ± 0.12	26.78 ± 0.34	7.39 ± 0.59	6.62 ± 0.35
F2F3 + P2	7.13 ± 0.43	45.0 ± 0.36	1.52 ± 0.17	3.50 ± 0.28	34.28 ± 2.05	4.28 ± 0.45	4.26 ± 0.37
EV	4.88 ± 0.52	41.75 ± 1.51	n.d.	2.0 ± 0.26	51.35 ± 2.29	n.d.	n.d.
FA composition of DAG (mol %)							
F2F3	12.17 ± 0.00	40.15 ± 0.52	n.d.	6.68 ± 0.57	40.99 ± 0.04	<0.01	<0.01
F2F3 + P1	14.54 ± 0.01	33.55 ± 1.33	n.d.	10.20 ± 0.60	28.88 ± 0.32	6.75 ± 0.33	6.06 ± 0.07
F2F3 + P2	13.35 ± 0.01	36.66 ± 0.09	n.d.	8.67 ± 0.45	34.32 ± 0.41	3.32 ± 0.12	3.68 ± 0.00
EV	10.19 ± 0.33	39.33 ± 0.53	n.d.	5.42 ± 0.23	45.05 ± 0.04	n.d.	n.d.
FA composition of TAG (mol %)							
F2F3	11.99 ± 0.06	51.58 ± 0.67	n.d.	4.04 ± 0.18	32.38 ± 0.55	<0.01	<0.01
F2F3 + P1	13.0 ± 0.12	42.29 ± 0.63	2.56 ± 0.28	6.56 ± 0.11	24.09 ± 0.55	4.57 ± 0.33	6.64 ± 0.35
F2F3 + P2	12.56 ± 0.07	45.58 ± 0.54	1.66 ± 0.04	5.66 ± 0.17	26.93 ± 0.72	2.75 ± 0.08	4.85 ± 0.16
EV	10.17 ± 0.15	49.17 ± 0.82	n.d.	4.03 ± 0.13	36.63 ± 0.53	n.d.	n.d.

^a^Data represent results from three independent yeast colonies (means ± SE).

^b^Not detected.

Fatty acid composition of phosphatidylcholine (PC), diacylglycerol (DAG) and triacylglycerol (TAG) from transgenic yeast expressing *LuFAD2* (F2) or *LuFAD3b* (F3) alone, or along with *LuPDCT1* (P1) or *LuPDCT2* (P2), as well as wild-type yeast transformed with empty pYESBOP vector (EV).

To further our understanding of the role of PDCT in PUFA accumulation, *LuPDCT1* and *LuPDCT2* were independently co-expressed with *LuFAD2* and *LuFAD3b* in WT yeast under the control of galactose inducible promoters (See Materials and Methods for details). Intriguingly, the combined level of LA and ALA increased approximately 6- and 4-fold in the PC fraction when *LuPDCT1* and *LuPDCT2* were co-expressed with the aforementioned desaturases, respectively (Table 1). Similarly, expression of the *LuPDCT* genes in this context also resulted in a substantial increase in the accumulation of PUFAs in both the TAG (12.8%) and DAG (11.2%) fractions (Table 1). Interestingly, a concomitant decrease in OA was observed in all three of the lipid fractions in both *LuPDCT1* and *LuPDCT2*-transformed cells (Table 1). Furthermore, an accumulation of 16:2^*cis*Δ9,12^ in both the PC and TAG fractions, but not DAG, were observed only in those cells harbouring the *LuPDCT* genes (Table 1).

### Expression of *LuPDCT1* and *LuPDCT2* restores the FA composition of the Arabidopsis *rod1* mutant

To provide additional evidence that *LuPDCT1* and *LuPDCT2* encode functional proteins, their corresponding open reading frames were expressed in the seeds of the *A. thaliana rod1* mutant, which exhibits a marked decrease in 18:2 and 18:3 PUFAs, along with a concomitant increase in 18:1 relative to WT plants [12,33]. Since genome position-related expression variation can occur in transgenic plants due to the random nature of transgenic insertions [34], we analyzed at least ten independent lines bearing each construct, respectively.

**Figure 5:**
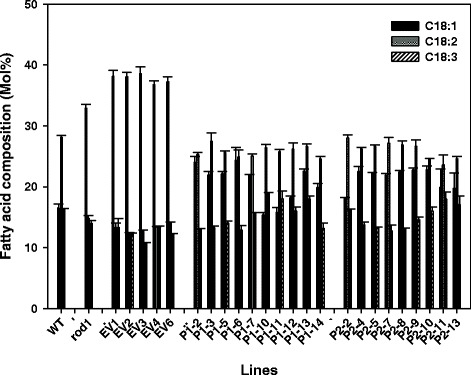
Expression of *LuPDCT1* or *LuPDCT2* restores the fatty acid composition of the *A. thaliana rod1* mutant. Fatty acid composition of the *A. thaliana rod1* mutant bearing *LuPDCT1* or *LuPDCT2* expression cassettes, respectively. Proportions of C18:1, C18:2, and C18:3 in untransformed wild-type seeds (WT), *rod1* mutant seeds (rod1), T_2_ seeds of the *rod1* mutant transformed with empty vector (EV), and T_2_ seeds of the *rod1* mutant bearing either *LuPDCT1* (P1) or *LuPDCT2* (P2) expression cassettes. Data shown represent means ± SE (*n* = 3).

Analysis of T_2_ -segregating transgenic seeds revealed that both LuPDCT1 and LuPDCT2 were capable of restoring the FA composition of the *rod1* mutant to WT levels in the majority of lines analyzed, whereas empty vector-transformed plants exhibited a FA profile similar to that of the *rod1* mutant (Figure 5; Additional file 1: Table S1). On average, expression of either *LuPDCT* gene caused the combined proportion of LA and ALA (C18-PUFAs) to increase to over 41% of the total FA content from approximately 27% in the *rod1* mutant, while OA levels decreased to nearly 21% from 33% (Additional file 1: Table S1). This corresponds well with wild-type OA, LA and ALA proportions, which make up 16.5%, 28.1% and 16.3% of the total FA content, respectively (Additional file 1: Table S1). This compensation of PUFA levels at the expense of OA further confirms that both *LuPDCT1* and *LuPDCT2* encode functional PDCT enzymes.

### Heterologous expression of *LuPDCT1* and *LuPDCT2* increases PUFA accumulation in wild-type *A. thaliana*

**Figure 6:**
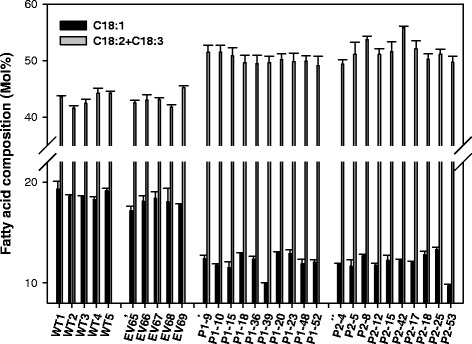
Heterologous expression of *LuPDCT1* or *LuPDCT2* increases polyunsaturated fatty acid (PUFA) content in wild-type (WT) *A. thaliana*. Fatty acid composition of wild-type *A. thaliana* bearing *LuPDCT1* or *LuPDCT2* expression cassettes. Proportions of C18:1 and C18-PUFAs (C18:2 + C18:3) in untransformed WT seeds, T_2_ seeds of WT *A. thaliana* bearing empty vector (EV), *LuPDCT1* (P1) or *LuPDCT2* (P2) expression cassettes. Data shown represent means ± SE (*n* = 3).

To gain insight into the role of PDCT in PUFA accumulation within seeds, and to determine whether the results we observed in a yeast system would translate to a plant system, *LuPDCT1* and *LuPDCT2* were independently expressed in WT *A. thaliana*. Analysis of T_2_-segregating seeds of independent transgenic lines bearing each construct, respectively, revealed significant changes in FA composition compared to negative control lines (WT and empty vector-transformed). On average, the proportion of C18-PUFAs increased from 43.1 ± 0.57% in empty vector-transformed seeds to 50.2 ± 0.26% and 51.6 ± 0.61% in *LuPDCT1* and *LuPDCT2* transformed seeds, respectively (Figure 6, Additional file 1: Table S2). Furthermore, the OA levels of the *LuPDCT*-expressing lines also dropped to approximately 12.0 ± 0.28% from 17.9 ± 0.21% in empty vector-transformed seeds (Figure 6, Additional file 1: Table S2), which supports the premise that PDCT plays a key role in PUFA production by enhancing the channelling of oleoyl moieties into PC for desaturation.

## Discussion

Flax seed is a rich agricultural source of ALA, and has been classified as one of the most important plant-based suppliers of PUFAs. Unfortunately, there is limited information concerning the metabolic pathway of oil synthesis in this species, which has significantly hindered its genetic improvement as an oilseed crop. However, a number of genetic and biochemical studies have been initiated using this species in recent years, and they are now beginning to generate insight into this previously unknown territory.

To date, it has been assumed that flax FA desaturases were the primary enzymes required for the determination of PUFA content in TAG. However, recent studies in *A. thaliana* have since demonstrated the importance of phosphocholine headgroup exchange between PC and DAG in providing PUFA for TAG synthesis as this mechanism appears to control the majority of acyl flux through PC [35]. PC is the enzymatic substrate for FA desaturases, and PC containing 18:1 at the *sn-*2 position is the initial substrate recognized by FAD2 leading to the production of C18-PUFAs [36]. Following desaturation by FAD2 and FAD3, the modified FAs are moved out of PC to eventually become incorporated into TAG. Thus, the efficiency of incorporation of FAs into PC and successive removal of desaturated FAs from PC for eventual TAG synthesis could be of great importance in determining the final FA composition of TAG in flax.

The two major pathways controlling the movement of FAs into and out of PC are acyl editing and DAG-PC interconversion via the action of PDCT after *de novo* synthesis of DAG through the Kennedy pathway. This symmetrical interconversion of PC and DAG liberates PC-derived DAG with modified FAs to be utilized by either DGAT or PDAT for TAG production. The ability of PDCT to catalyze the symmetrical interconversion between DAG and PC through a phosphocholine headgroup exchange was first demonstrated in plants through the characterization of the *A. thaliana rod1* mutant [12]. PDCT was found to act as a gatekeeper enzyme, providing a major path through which oleoyl moieties entered PC for desaturation at the *sn*-2 position. This mutation resulted in a significant decrease in PUFA levels in seed TAG compared to WT (29.4% vs. 49.1%, respectively) [12], demonstrating the importance of PDCT in the efficient production of PUFAs in this species. Indeed, in Arabidopsis seeds it has been estimated that at least 40% of PUFAs in TAG are derived through the action of PDCT alone [2]. Furthermore, it has recently been shown that the collective action of PDCT along with acyl-CoA:lysophosphatidylcholine acyltransferases (LPCATs) are responsible for the accumulation of nearly two-thirds of the C18-PUFAs in this species [35]. Recently, it has also been demonstrated that PDCT is required for efficient hydroxy fatty acid accumulation in transgenic *A. thaliana* [14], demonstrating the importance of PDCT in channelling modified fatty acids into TAG.

While PDCT has been shown to play a key role in the production of TAG enriched with modified FAs, including PUFAs, in model plant species, this is the first study in which the importance of this enzyme from a crop plant specifically grown for its high levels of PUFAs has been analyzed. Interestingly, results from a number of recent studies have suggested that Arabidopsis is not the only plant species that utilizes PC-derived DAG to produce PUFA-rich TAG or TAG enriched with other modified FAs (epoxy-, conjugated-, hydroxy-, acetylenic-FAs) [13]. For example, it has been estimated that flax utilizes more than about 70% PC-derived DAG to synthesize TAG [13-37], which implies that the PDCT-catalyzed reaction may be the main route of PC-derived DAG synthesis in flax and may therefore be an important contributor to the high levels of PUFAs found in this crop species.

In an initial attempt to provide evidence that this is, indeed, the case, we have isolated cDNA from two flax *PDCT* homologs (*LuPDCT1* and *LuPDCT2*) that displayed high levels of sequence identity with one another (97% and 98% at the nucleotide and amino acid levels, respectively; Figure 1). Flax contains a number of highly homologous gene pairs encoding enzymes involved in oil biosynthesis [24], which is consistent with our results and may be the consequence of a putative whole-genome duplication that has been suggested to have occurred in this species [20]. Phylogenetic comparison of the two LuPDCT proteins with other higher plant PDCTs, including functionally tested enzymes from *A. thaliana* [12] and castor [14], indicated that they are more closely related to homologous proteins from plants such as spruce and castor than they are to that from *A. thaliana* (Figure 1B). This has also been found to be the case for several other flax oil biosynthetic genes [24]. The fact that the PDCT homologs with the highest sequence identity to the two LuPDCT proteins tended to be from other high C18-PUFA and unusual FA-producing plants such as sunflower (*Helianthus annuus*), cotton (*Gossypium hirsutum*) and castor (*Ricinus communis*) (Additional file 1: Figure S1) suggests that PDCT likely provides an important function in the incorporation of modified FAs into TAG in many plant taxa. Interestingly, PDCT homologs have not been found to be present in certain plant species that accumulate conjugated FAs, including bitter melon (*Momordica charantia*) and tung tree (*Vernicia fordii*) [38], which implies that PDCT may not be ubiquitously distributed throughout the plant kingdom. It would be interesting to compare flax PDCT with chia (*Salvia hispanica*) PDCT, if homologs are present, once the sequence information becomes available as chia has a close C18-PUFA profile to flax [39].

As is the case for *AtROD1* [12], *LuPDCT1* and *LuPDCT2* transcript levels were dramatically higher in developing flax embryos than in other parts of the plant (Figure 2). This increase in transcript levels, which peaked at approximately 11 DAA and declined to nearly undetectable levels by 16 DAA (Figure 2), correlates well with the timing of rapid oil (and ALA) accumulation in developing embryos of this species [24] and provides strong evidence that the LuPDCT proteins are involved in the production of high levels of C18-PUFAs in flax. We also noted very low levels of *LuPDCT1* and *LuPDCT2* transcripts in vegetative tissues and flowers (Figure 2), which is in contrast to the expression of *AtROD1* in these tissues in *A. thaliana* [12]. These findings imply that the LuPDCT enzymes may provide a secondary function of PUFA enrichment in non-oil-accumulating tissues in addition to TAG-rich embryos in flax. In agreement with this, the *rod1* mutation in *A. thaliana* did not result in substantial FA compositional changes in leaf or root whereas PUFA levels were substantially decreased in seeds [12,33].

Enzyme assays with microsomal preparations from *S. cerevisiae* cells expressing *LuPDCT1* or *LuPDCT2* demonstrated that both proteins possessed PDCT activity in catalyzing both forward and reverse reactions (Figures 3 Additional file 1: Figure S2). When assayed for the formation of DAG from PC, a small quantity of TAG was also produced (Additional file 1: Figure S3), which likely resulted from the catalytic action of yeast TAG synthesizing enzymes present in the microsomal fraction. Interestingly, previous studies indicated that both AtROD1 [12] and RcROD1 [14] exhibited high PDCT activities at 15°C; however, both LuPDCT1 and LuPDCT2 exhibited extremely low activity at 15°C compared to 30°C (data not shown). Although enzymatic activity data was not available for AtROD1 and RcROD1 at 30°C and different enzyme assay conditions were utilized in the various studies, these data indicate that the flax PDCT proteins may possess different enzymatic properties than those of *A. thaliana* or castor.

Direct evidence for the role of LuPDCT enzymes in C18-PUFA enrichment was obtained through the expression of the corresponding genes in *FAD2/FAD3*-expressing yeast, where major compositional changes were observed in the different lipid classes compared to yeast lacking the *LuPDCT* coding sequences (Table I). Metabolic engineering of yeast with *LuFAD2* and *LuFAD3b* expression cassettes provided an excellent system with which to study the effects of *PDCT* expression on PUFA accumulation in PC, DAG and TAG fractions without having to resort to the feeding of yeast cultures with LA and ALA, which are not produced naturally by *S. cerevisiae*. FA feeding in an attempt to introduce exogenous acyl moieties to yeast cells is often utilized in such situations, but FA uptake into yeast cells can at times lead to deleterious effects [40], making the system developed here a much more desirable option.

When only the *FADs* were expressed, PUFAs were observed in the PC fraction, with only minute quantities being present in DAG and TAG fractions. Co-expression with each *LuPDCT*, however, resulted in a marked increase in PUFA levels in both DAG and TAG fractions (up to 12.81% and 11.21%, respectively; Table 1), providing further support for the premise that PDCT plays a critical role in channelling PUFAs to DAG, and subsequently TAG, following production on PC. Moreover, C18-PUFA content in the PC fraction was also substantially increased (a 5-fold increase for LuPDCT1 and 8.5-fold increase for LuPDCT2; Table 1) at the concomitant expense of 18:1. Taken together, these data suggest that PDCT enhances the channelling of oleyol acyl moieties into PC by rapidly converting DAG to PC, thus increasing substrate availability for FADs. This dual mechanism of PDCT (converting PUFA-enriched-PC to PUFA-enriched-DAG to be used for TAG production, and converting DAG with oleyol acyl moieties to PC, thus generating more substrate for FADs) appears to result in a synergy leading to increased PUFA accumulation in this yeast system.

These results were confirmed in a plant system, wherein both wild-type and *rod1* mutant *A. thaliana* were transformed with vectors designed to express *LuPDCT1* and *LuPDCT2*, respectively. In the case of the transgenic *rod1* mutant plants, either *LuPDCT1* or *LuPDCT2* was capable of restoring PUFA levels to more WT levels (Figure 5). There was some variation in the level of complementation of the FA composition in the transgenic lines (Figure 5; Additional file 1: Table S1), which was likely due to positional effects of transgene insertion; this is a common phenomenon in the generation of transgenic plants. Similar variations among independent transformants were observed when the castor *FATTY ACID HYDROXYLASE* gene (*CFAH12*) was expressed in the *A. thaliana fad2/fae1* double mutant [41] or fatty acid elongase (*FAE*) gene from nasturtium (*Tropaeolum majus*) was overexpressed in WT *A. thaliana* [42].

In line with results obtained with the *rod1* mutant, expression of the two flax *PDCT* genes in WT *A. thaliana* substantially increased PUFA production in transgenic lines. On average, the proportion of C18-PUFA increased by 16.4% and 19.7% in *LuPDCT1*- or *LuPDCT2*-expressing lines, respectively, at the concomitant expense of 18:1 (Figure 6, Additional file 1: Table S2). These data provide further support that PDCT increases PUFA accumulation by enhancing the channelling of monounsaturated acyl moieties through desaturation on PC and making available the resulting polyunsaturated acyl moieties for TAG synthesis. Compositional analysis of flax seed lipids has previously revealed that approximately 95% of its TAG is enriched with PUFAs at any one of the *sn*-positions, whereas only approximately 40% of its TAG contains C18-PUFAs at all three *sn*-positions [43], indicating that there is a vast potential to increase the proportion of PUFA-rich TAG in this species.

## Conclusions

We have identified two novel *PDCT* homologs from flax, which encode enzymes catalyzing increases in C18-PUFA levels substantially in metabolically engineered yeast and transgenic *A. thaliana* seeds. These flax PDCTs appear to play an important dual role in the determination of PUFA content by efficiently channelling monounsaturated FAs into PC for desaturation and moving the resulting PUFAs out of PC for subsequent use in TAG synthesis. These results have distinguished *PDCT* as an ideal candidate gene for future bioengineering of flax and other oil crops to produce improved levels of PUFAs for widespread use in industrial and nutraceutical applications.

## Methods

### Plant materials

Flax plants (cultivar CDC Bethune) were grown in a Conviron growth chamber (http://www.conviron.com) at 25°C/17°C (day/night) with a 16-/8-h photoperiod under cool-white fluorescent lights (F54/I5/835/HO, high fluorescent lights, Phillips, Holland; 360 μE m^−2^ s^−2^). Vegetative tissues, including the five most immature leaves, three sections of stem, apices, and roots, were harvested from four-week-old flax seedlings. In the case of stem tissue, the ‘immature’ stem sample contained the three most immature internodes, the ‘developing’ stem sample contained the next two developing internodes, and the ‘mature’ stem sample contained the two most mature internodes. Flowers were collected at the approximately half open stage. For collection of developing embryos, flowers were individually tagged on the day of anthesis, which was considered day 0. Developing and mature bolls were then harvested at 4, 8, 11, 14, 16, 20, 25, and 40 DAA, and embryos/seeds were subsequently collected. Due to technical difficulties in separating embryos from other seed components at 4, 25 and 40 DAA, whole seeds were used for gene expression analysis rather than embryos at these three stages. All tissue was immediately frozen in liquid nitrogen following collection and stored at −80°C.

### RNA extraction and cDNA synthesis

Total RNA was extracted from vegetative tissues, flowers and developing embryos using the RNeasy Plant Mini Kit (Qiagen, http://www.qiagen.com), while the Spectrum Plant Total RNA Kit (Sigma, http://www.sigmaaldrich.com) was utilized for mature seeds (25 and 40 DAA). Following extraction, RNA samples were treated with the DNA-free kit (Ambion, http://www.lifetechnologies.com/ambion) to remove any residual DNA. RNA integrity was verified by determination of the 260 to 280 nm ratio by spectrophotometry, as well as gel electrophoresis. RNA concentration was determined using a NanoDrop ND-100 spectrophotometer (NanoDrop Technologies, Wilmington, DE). First-strand cDNA synthesis was performed in a final volume of 20 μL using 0.5 μg of total RNA as template and the QuantiTect reverse transcription kit (Qiagen) according to the manufacturer’s instructions.

### Cloning of *PDCT* genes from flax

The *AtROD1* sequence (At3g15820) [12] was used to query the flax genomic sequence database (www.linum.ca) [20] using the basic local alignment search tool (BLAST) [21]. Analysis of the resulting alignments of positive hits identified two *PDCT* genes in the flax genome. The following primers pairs bearing restriction sites near their 5′ ends to facilitate subsequent cloning were used to amplify full-length cDNA sequences from both genes using cDNA preparations from developing embryos. FP1: 5′ - ATA T*GG ATC C*TA CAC AAT GTC TGC CGC ACC CAC CAC CAC CGT - 3′ (*BamHI* is underlined), RP1: 5′ - ATA T*AA GCT T*CT AGT TGG TGA CAA ATT TTC TGG GAG AA - 3′ (*HindIII* is underlined) and FP2: 5′-ATA T*GG ATC C*TA CAC AAT GTC TGC CAC ACC CAC CGC CGC CGT - 3′ (*BamHI* is underlined), RP2: 5′ - ATA T*AA GCT T*CT AGT TGG TGA CAA ATT TTC TGG GAG AGG - 3′ (*HindIII* is underlined). The resulting amplicons were then cloned into *BamHI* and *HindIII* sites of the pYESBOP expression vector, which is a modified version of the pYES2.1/V5-HIS vector [24], and sequenced to confirm their identities. The resulting recombinant vectors were designated pYES-PDCT1 and pYES-PDCT2.

### Phylogenetic analysis

Sequences of PDCT-like proteins (Additional file 1: Figure S1) were obtained from the sources indicated in Additional file 1: Figure S1. Sequence alignments were generated using ClustalW through the Geneious v5.3 (www.geneious.com) software using the default parameters, including a Gonnet scoring matrix, a gap penalty of 10 and gap length penalty of 0.2. The phylogenetic tree was constructed using the same software with default parameters, including the neighbor-joining tree building method, Jukes-Cantor genetic distance method and no outgroup.

### qRT-PCR

Quantification of *LuPDCT1* or *LuPDCT2* transcripts in various flax tissues was performed using 7.2 μL of a 1/40 dilution of cDNA as template with Taqman Fast Advance Master Mix (Applied Biosystems, http://www.appliedbiosystems.com) in a final volume of 25 μL. Primers (300 nM) and Taqman probes (10 nM) were designed to specifically anneal to *LuPDCT1*, *LuPDCT2*, or the two reference genes, *GLYCERALDEHYDE-3-PHOSPHATE DEHYDROGENASE* (*GAPDH*) and *UBIQUITIN EXTENSION PROTEIN* (*UBI2*), which have been shown previously to be expressed stably in flax [44] (for sequences, see Additional file 1: Table 3). PCR efficiencies for each primer pair were calculated using a dilution series of a single cDNA sample over several log concentrations. All reactions were carried out using the ABI PRISM 7900 HT Real Time PCR system (Applied Biosystems) with the following thermal parameters: 50°C for 2 min, 95°C for 20 s and 40 cycles of 95°C for 1 s and 60°C for 20 s. Primer specificity was verified by separating resulting qRT-PCR amplicons on a 2% agarose gel. Relative levels of *LuPDCT1* or *LuPDCT2* transcripts were calculated using the comparative C*t* method after normalizing to the two reference genes. The average of three biological and technical replicates, respectively, was used to denote transcript abundance in each case.

### Heterologous expression of flax *LuPDCT* cDNAs in *S. cerevisiae*

The *S. cerevisiae* mutant strain YNL 130C (*MAT*α *cpt1:: KanMX ept1)*, which lacks CPT activity [25], was obtained from Openbiosystems (Huntsville, AL) and was transformed with pYES-PDCT1, pYES-PDCT2 and empty pYESBOP as a negative control according to Gietz and Schiestl (2007). Following transformation, yeast was grown in minimal medium containing 2% (w/v) raffinose as described by Siloto et al. [40]. Galactose-induced expression of the recombinant *LuPDCT* cDNAs was performed in yeast nitrogen base (YNB) medium containing 2% (w/v) galactose and 1% (w/v) raffinose.

#### *In vitro* PDCT assays

For the preparation of microsomes, yeast cultures were grown in induction medium for 20 h as described above, and membrane fractions were isolated according to Siloto et al. [40] with the exception that for homogenization, ice-cold glucose-Tris-EDTA (GTE) buffer [20% glycerol, 50 mM Tris–HCl (pH 7.4), 1 mM EDTA] was used [12]. The protein content of the microsomal fractions was determined using the Bradford assay (BioRad, http://www.bio-rad.com) with bovine serum albumin as the standard.

PDCT assays were performed essentially as described by Lu et al. [12], with some modifications. For assays in the direction of radio-labeled PC formation, 0.5 nmol of [^14^C]-glycerol- *sn*-1,2-diolein (55 μCi/μmol; American Radiolabeled Chemicals, St. Louis, MO), 2.5 nmol of unlabeled *sn*-1,2-diolein, and 100 nmol of dioleoyl-PC were mixed and dried immediately under nitrogen gas. For assays in the direction of radio-labeled DAG formation, 2.5 nmol of *sn*-1-palmitoyl-*sn*-2-[^14^C]oleoyl-PC (55 μCi/μmol; American Radiolabeled Chemicals), 100 nmol of diolein and 10 nmol of unlabeled *sn*-1-palmitoyl-*sn*-2-oleoyl-PC were mixed and dried immediately under nitrogen gas. Dried residues were then resuspended in 50 μL of 4X reaction buffer [200 mM 3-(N-morpholino) profanesulfonic acid/NaOH (pH 7.5), 80 mM MgCl_2_, 1.8% Triton X-100] using a sonication bath. Enzyme reactions were initiated by adding 100 μg of microsomal protein (in 150 μL GTE buffer) and allowed to proceed at 30°C for different incubation times for assays in the direction of radio-labeled PC formation. Assays in the direction of radiolabeled DAG formation were allowed to proceed for 15 min. Reactions were quenched with 3 mL chloroform/ethanol (2:1) and 1.5 mL of 0.9% KCl. The lower organic phase was then extracted, dried under nitrogen gas and resuspended in 80 μL chloroform. The resulting extracts were then subjected to TLC on silica gel plates (SIL G-25, 0.25 mm, Macherey-Nagel, Germany), which were subsequently developed in chloroform/methanol/acetic acid/water (60:30:3:1,v/v/v) followed by hexane/diethyl ether (80:20, v/v). The amount of [^14^C]dioleoyl-PC and 1-palmitoyl-2-[^14^C]-oleoyl-DAG produced was monitored by phosphor-imaging analysis (Typhoon multi-purpose imager; GE Healthcare, Mississauga, ON), and the appropriate bands were scraped off the plates and subjected to scintillation counting to determine radioactivity (Beckman-Coulter 6500 Liquid Scintillation Counter, Mississauga, ON).

### Engineering *S. cerevisiae* to produce PUFAs

*LuFAD2-* and *LuFAD3b*-specific primers were designed bearing restriction sites near their 5′ ends to facilitate cloning based on GenBank accession numbers DQ222824 [30] and DQ116425 [28], respectively, and the corresponding coding regions were amplified by PCR using cDNA preparations derived from developing embryos of flax cultivar CDC Bethune. The full-length *LuFAD2* coding region was amplified using primers FAD2F [5′- ATA *GGA TCC* ACC ATG GGT GCT GGT GGA AGA AT −3′ (*BamHI* site is underlined)] and FAD2R (5′- TAT *GGT ACC* TCA CAG CTT GTT GTT GTA CCA - 3′ (*KpnI* site is underlined)], while *LuFAD3b* was amplified using primers FAD3F [5′ - CCG *GAA TTC* TAC ACA ATG TCA ATG AGC CCT CCA AAC TCA ATG - 3′ (*EcoRI* site is underlined)] and FAD3R [5′ - TAT *GAG CTC* TCA GCT GGA TTT GGA CTT GG - 3′ (*SacI* site is underlined)]. The resulting *LuFAD2* or *LuFAD3b* amplicons were subsequently cloned into the corresponding restriction sites of the pESC-URA yeast expression vector (Agilent, http://www.agilent.com). The entire *LuFAD2* or *LuFAD3b* expression cassettes were then amplified and cloned into pYES-LuPDCT1, pYES-PDCT2, and empty pYESBOP vectors, respectively, via phosphorothioate-based ligase-independent gene cloning (PLICing) [45,46] using primers PL-F (5′ - A*C*C* A*T*G* G*G*C* A*G*C* GAG CGA CCT CAT GCT ATA CCT GA - 3′) and PL-R (5′ - G*G*C* T*T*T* G*T*T* A*G*C* CTT CGA GCG TCC CAA AAC CTT CT - 3′). Recombinant vectors were termed pYESF2F3 + P1, pYESF2F3 + P2 and pYESF2F3, respectively, and were separately transformed into WT *S. cerevisiae* according to Gietz and Schiestl [47]; empty pYESBOP vector was also transformed into yeast as a negative control.

### FA analysis of transformed yeast lipids

Transformed yeast cultures derived from three colonies in each case were grown in minimal medium containing 2% (w/v) raffinose as described by Siloto et al. [40]. Galactose-induced expression of the recombinant genes was performed using yeast nitrogen base (YNB) medium containing 2% (w/v) galactose and 1% (w/v) raffinose in which cultures were grown at 20°C with shaking at 225 rpm for 3 days. Total lipids were extracted from induced yeast cells according to the method of Bligh and Dyer [48]. The internal standards 1, 2-dinonadecanoyl-*sn*-glycero-3-phospcholine (19:0-PC), 1, 2-dipentadeconyl-*sn*-glycerol (15:0-DAG) and triheptadecanoin (17:0-TAG) were added to each sample to facilitate visualization of the three different lipid fractions on TLC plates. Total lipid extracts were separated by 1D TLC on silica gel plates (SIL G-25, 0.25 mm, Macherey-Nagel, Germany), which were subsequently developed in chloroform/methanol/acetic acid/water (60:30:3:1) followed by hexane/diethyl ether (80:20). Lipids were visualized on the TLC plates using UV illumination after spraying with 0.05% premuline solution. Spots corresponding to TAG, DAG and PC were scraped off the plates and transmethylated with 5% sodium methoxide at room temperature for 30 min. FA methyl esters were extracted with hexane and dried under nitrogen gas. Fatty acid methyl ester extracts were then resuspended in 0.5 mL of iso-octane including C21:0 (methyl heneicosanoin (0.1 mg/mL)) as a standard and subsequently analyzed by gas chromatography–mass spectrometry as described by Mietkiewska et al. [49].

#### ***A****. Athaliana* transformation and vector construction

The full-length coding regions of *LuPDCT1* and *LuPDCT2* were amplified by PCR and cloned downstream of the napin promoter and upstream of the rubisco transcriptional terminator between *BamHI* and *XbaI* sites of the pGreen0229 vector [50]; (http://www.pgreen.ac.uk). The resulting vectors were electroporated into *Agrobacterium tumefaciens* strain GV3101 [51] along with the helper plasmid, pSOUP. Plasmid identities were verified by DNA sequencing following re-isolation from *Agrobacterium* and transformation into *E. coli*. Wild-type Arabidopsis (ecotype col-0), as well as the *rod1* mutant line DH4 [12,33], were transformed with *LuPDCT*-containing vectors, along with empty pGreen0229 vector as a negative control, using the floral dip method [52]. Resulting T_1_ seeds were screened on half-strength Murashige & Skoog medium [53] supplemented with 80 μM phosphinothricin. Transgenic *A. thaliana* seedlings were transferred to soil and grown in a growth chamber to maturity with a day/night temperature of 22/20°C and a photoperiod of 16 h light and 8 h dark. The presence of the *LuPDCT* cassettes was confirmed in each case by gene-specific PCR analysis using DNA extracted from leaf tissue as template. T_2_ seeds were collected from independent transgenic lines bearing each construct, respectively, and used for subsequent FA analysis.

### FA analysis of *A. thaliana* seed oil

The FA profile of three technical replicates of T_2_ seeds of independent transgenic lines bearing *LuPDCT1* and *LuPDCT2* expression cassettes, along with the empty vector control, respectively, was determined by gas chromatography–mass spectrometry using the method described by Pan et al. [24].

### Availability of Supporting Data

Sequence data from this article can be found in the GenBank/EMBL data libraries under accession numbers KC669705, KC669706, KC469054 and KC469055. Aligned sequence data and phylogenetic trees were deposited in TreeBASE under the Study Accession Number S17477.

The data sets supporting the results of this article are included within the article as following supplementary figures and tables.

## Additional File

Additional file 1:
**Figures S1-S3, Table S1-S3.**

## Abbreviations

ALA: α-linolenic acid
CPT: CDP-choline diacylglycerol cholinephosphotransferase
DAG: diacylglycerol
DGAT: diacylglycerol acyltransferase
FA: fatty acid
FAD: fatty acid desaturase
GTE: glucose-Tris-EDTA
LA: linoleic acid
LPCAT: lysophosphatidylcholine acyltransferase
OA: oleic acid
PC: phosphatidylcholine
PCR: Polymerase chain reaction
PDAT: phospholipid:diacylglycerol acyltransferase
PDCT: phosphatidylcholine diacylglycerol cholinephosphotransferase
qRT-PCR: Quantitative real-time PCR
TAG: triacylglycerol
TLC: thin layer chromatography
PUFA: polyunsaturated fatty acid
WT: wild-type
