# Supplementary material for: Heterologous expression of flax PHOSPHOLIPID:DIACYLGLYCEROL CHOLINEPHOSPHOTRANSFERASE (PDCT) increases polyunsaturated fatty acid content in yeast and Arabidopsis seeds
Source: BMC Biotechnol. 2015 Jun 30;15:63. doi: 10.1186/s12896-015-0156-6 (PMC4486708; doi:10.1186/s12896-015-0156-6)
Supplement: Additional file 1: — Figures S1-S3, Table S1-S3. [file 12896_2015_156_MOESM1_ESM.pdf]

Additional Files

```
LuPDCT1             ------MS-AAPTTTVAATAIPLKRKKTDA------------------------------
LuPDCT2             ------MS-ATPTAAVAATAIPLKRKKTDA------------------------------
AtROD1              ------MS-----AAAAETDVSLRRRSNSLNGNHTNGVA-------IDGTLDNNNRRVGD
RcROD1              ------MKSTVPPTTTTTTTTTLYKRKKDINLTSVNDSV-----DMVS-----NKN----
Brassica            --------------MSTNTVVPLRRRS---NGYHTNGVA-------FNG-----------
Brachypodium        ---------MPPPSLAAAQDQVAANAA--G-----NGIDARRRAGKGK--------KVHP
Gossypum            ------MKTAEATAATTTVLSRGYKRSPKS-----NG------------------KVNGT
Glycin              ----------------------------------MNGGA-----EASV-----NHRRRHQ
Hordeum             ---------MPPASLTAAHDRASAANG--L-----AGPETRRRAGKGK--------KVHP
Medicago            ----------------------------------MNGGA-----ASSSGDATVLKRRHTT
Oryza               ------MPPPPPPSLTANTASSMGNAE--AVVVLPANGGARRRADKVV--------HPAP
Picea               --------------------MSCRRRSSEDNGGETDMIYVDRRTKQCLGNLFCICSQEIQ
Populus             ----------------------------------------------ME-----NKN----
Sorghum             ---------MPPPSLTAAAATTT---------------TRRRKD--------------HP
Helianthus          MTVEKLHHRTVPQTTTT-----ILQPPNNIS-----------------------------
Zea                 ---------MPPPSLTAAGTTTT---------------TTRRRNDRAA--------KVHQ


LuPDCT1             -AA-KTQKNNQG-----NEDEQEAIVESVAPAFTKWRRRDAMNAVREHWLPVILGGALLF
LuPDCT2             -AA-KTQKNNQG-----NEDEQEAIVESVAPAFTKWRRRDAMNAVREHWLPVMLGGALLF
AtROD1              TNTHMDISAKKTD-NGYANGVGGGG-WRSKASFTTWTARDIVYVVRYHWIPCMFAAGLLF
RcROD1              -F--ANGNVNGG--------GGYTAFNRFDPSFMKWTTHDVVNVVKFHWLPCVFGLGLLF
Brassica            ----MDNIVKKTD-DCYTNGNGNGGVERSKASFLTWTMRDAVYVARYHWIPCFFAVGVLF
Brachypodium        LPDAG---TTMGD-GVGMGG---GESRKGRPEWM--TPAGAAGVLWRHPLPVVFACGLLL
Gossypum            AA--KKTGVNHG---------NGGDISNGKASFMKWTLDDVVYVAKYHRLPCFFAAWFLF
Glycin              AA--SANGVKIAN-GAM---AKPSSTLCYDASFMKWTVADAVHVATHHWMPCLFALGLLF
Hordeum             LPPDG---AAMGD-GGERLA---GG--RRPADWL--SPSGVAGILRRHPLPALFACGLLL
Medicago            TN--QTNHSNQKN-PMPKNSSSSLCSLYSSASFLKWTISDVVHVATHHWMPCLFACGLLF
Oryza               MPDRAAGGAMERE-GGGVGGGGEVGGW-RRPEWC--SAAGVAGVLRRHPAAAAFGCGLLL
Picea               RKLRDLMGANGNVVHQDEKSPFPLGDTAVYPSFMGWSMTYLIGTARFHPLPVLLVGCLLF
Populus             -K--QTNGITTD--------GAANGFYGVDPSFLKWTLRDVVNVAKHHWLPCFLGFGLLF
Sorghum             APG---GGAGAKE-MGAAAA-SAAEGWARRPEWC--SAAGVAGVLRRHPAPALFGCGLLL
Helianthus          -----INKINLKNTHNHETTTTFSHRWSFDAFCLRWMVADVFGLLKYHPVPCFFGVSLLF
Zea                 VLG---EGAGTEE-MGAV-----ADGWT-RPEWC--SAAGVAGVLRRHPAPALFGCGLLL
                                                                   *          *

LuPDCT1             FMWVEYTLRMVPTSSQPFDLGFVATRALHRLLSSSPELNSVLAALNTVFVGMQTSYILWT
LuPDCT2             FMWVEYTLRMVPTSSQPFDLGFVATRALHRLLSSSPELNSVLAALNTVFVGMQTSYILWT
AtROD1              FMGVEYTLQMIPARSEPFDLGFVVTRSLNRVLASSPDLNTVLAALNTVFVGMQTTYIVWT
RcROD1              FMAVEYTLRMVPASSPPFDLGFLVTRHLHLLLSSWPALNTLLAFLNTVFVLMQTAYILWT
Brassica            FMGVEYTLQMVPAKSEPFDIGFVATRSLNRVLASSPDLNTLLAALNTVFVAMQTTYIVWT
Brachypodium        FMGVEYTIPMVPHAAPPLDLGFLATAAMHDGIAARPWLNSLLAALNTVFVAMQTAYILWA
Gossypum            FIYVEYTLRMVPDSSPPFDLGFVVTRSFHRALASWPELNSLLAALNTVFVAMQSVYIIGT
Glycin              FMAVEYTLLMVPPSSPPFDLGFIATRSLHALLESSPNLNTLFAGLNTVFVGMQTSYILWT
Hordeum             FMGVEYTIPMIPAAAPPLDLGFHATAAMHAGIAARPWLNSLLAALNTVFVAMQAAYILWA
Medicago            FMAVEYTLFMVPSSSPPFDLGFVFTRSLHRVLESSPQLNNVLAALNTVFVLMQTSYILWT
Oryza               FMAVEYTIPMVPPAAPPVDLGFAATAALHAGIAARPWLNSLLAALNTVFVAMQAAYILWA
Picea               FMAVEYTLVMVPAGSQPYDVGFVWTQSLHDLLLERPALNTVLAAMNTVFVGMQTFYILWT
Populus             FMAVEYTLRMVPSSSPPFDLGFVVTRRLHGLLSSWPELNTSLAGLNTVFVGMQTAYILWT
Sorghum             FMAVEYTIPMVRPDSPPLDLGFIATRNMHAAVAATPWLNSLLAALNTVIVAMQAAYILWA
Helianthus          FMGVEYTLWMVPSSSPPFDIGFVATAWLHRVLAASPVLNTVLAGLNTVFVGMQTTYILWT
Zea                 FMAVEYTIPMVKPDAPPLDLGFLATAGMHAAIAARPWLNSLLAALNTVFVAMQAAYILWA
                    *  ****  *      * * **  *          * **   *  *** * **  **

LuPDCT1             FVVEGRGRPTISALFMFTCRGILGYSTQLPLPEGYLGSGVDFPVGNVSFFLFFSGHVAGS
LuPDCT2             FVVEGRGRPTISALFMFTCRGILGYSTQLPLPEGYLGSGVDFPVGNVSFFLFFSGHVAGS
AtROD1              WLVEGRARATIAALFMFTCRGILGYSTQLPLPQDFLGSGVDFPVGNVSFFLFFSGHVAGS
RcROD1              WLIEGRPRATISALFMFTCRGILGYSTQLPLPEGFLGSGVDFPVGNVSFFLFFSGHVAGS
Brassica            WLMEGRPRATISACFMFTCRGILGYSTQLPLPQDFLGSGVDFPVGNVSFFLFYSGHVAGS
Brachypodium        ILAEQRPRAAIATLMMFTCRGLLGCSTQLPLPAEFLGSGMDFPVGNVSFFLFYSGHVAGA
Gossypum            WVVEGRPRATISALFMFTCRGILGYSTQLPLPQEFVGSGMDFPVGNVSFFLFYSGHVAGS
```

```
Glycin            WLIEGRPRATISALFMFTCRGILGYSTQLPLPQGFLGSGVDFPVGNVSFFLFFSGHVAGS
Hordeum           VLAEQRPRAAIATLMMFTCRGLLGCSTQLPLPAEFLGSGMDFPVGNVSFFLFYSGHVAGA
Medicago          WFIEGRPRATISALFMFTCRGICGYSTQLPLPQGFLGSGVDFPVGNVSFFLFFSGHVAGS
Oryza             ILGEGRPRAAVAAMMMFTCRGALGCATQLPLPAEFLGSGMDFPVGNVSFFLFFSGHVAGA
Picea             CVVEGRGRPTIAALFMFTCRGILGYTTQLPLPEEFLGSGVDFPVGNVSFFLFFSGHVAGA
Populus           WLVEGRPRATISALFMFTCRGILGFSTQLPLPEEFLGSGADFPVGNVSFFLFFSGHVAGS
Sorghum           ILAEQRPRAAVAALMMFTCRGVLGCATQLPLPEEFLGSGMDFPVGNVSFFLFFSGHVAGA
Helianthus        WVVEGRPRATVATLFMFTCRGILGYSTQLPLPEEFLGSGADFPVGNVSFFLFFSGHVAGS
Zea               ILAEQRPRAAVAALMMFTCRGVLGCATQLPLPEEFLGSGMDFPVGNVSFFLFFSGHVAGA
                     * * *       ******  *  ******    *** ************ ******

LuPDCT1           VIASRDMRRMQRWELALGFEFLNALQVVRLLATRGHYTIDLAAGYAAGLLFDSLAGRYLQ
LuPDCT2           VIASQDMRRMQRWELALGFEFLNALQVVRLLATRGHYTIDLAAGYAAGLLFDSLAGRYLQ
AtROD1            MIASLDMRRMQRLRLAMVFDILNVLQSIRLLGTRGHYTIDLAVGVGAGILFDSLAGKYEE
RcROD1            VIASLDMRRMQRWELAWTYDVLNVLQAVRLLGTRGHYTIDLATGVGAGILFDSLAGKYEE
Brassica          MIASLDMRRMQRLRLAMLFDILNILQSIRLLGTRGHYTIDLAVGVGAGILFDSLAGKYEE
Brachypodium      VIAAADMRRVGRLRLAALYDALNLIQGVRLLACRGHYTIDLAVGVGAGLLFDMLAGRYLD
Gossypum          VIASLDMRRMQRWELAWLFDILNVLQAVRLLGTRGHYTIDLAVGVGAGILFDSLAGKYIE
Glycin            VIASLDMRRMQRWELAWTFDVLNVLQAVRLLGTRGHYTIDLAVGVGAGILFDSLAGKYED
Hordeum           VIASIDMRRVGRRRLAALYDALNLAQGVRLLACRGHYTIDLAVGVGAGLLFDMLAGWYLD
Medicago          VIASLDMRRMNRWGLAWTFDLLNVLQAVRLLGTRGHYTIDLAVGLGAGYIFDSLAGKYLE
Oryza             VIAAEDMRRAGRRGMARLYDALNLLQGVRLLACRGHYTIDLAVGVGAGLLFDMLAGRYLD
Picea             VIASLDMRRVKRNQLAFTFDTLNALQSVRLLGTRGHYTIDLVAGVGAGWLFDSLAGKYEE
Populus           LIASLDMRRMQRWELARAFDLLNVLQVIRLLGTRGHYTIDLAVGVGAGVLFDSLAGKYVE
Sorghum           VIAAADMRREGRAALARLYDALNVLQAVRLLACRGHYTIDLAVGVGAGVLFDTLSGWYFD
Helianthus        GIASLDMRRMRRWELAFLFDTLNLLQVVRLLSTRGHYTIDLAIGVGAGMLIRFSCREIHE
Zea               VIAAADMRREGRLALARLFDSLNVLQVVRLLACRGHYTIDLAVGVGAGILFDTLSGWYFD
                   **  ****  *   *     **  *  ***  ********  *  **

LuPDCT1           TKTATAAALLTT------SPRKFVTN---------
LuPDCT2           TKTATAAALLTT------SPRKFVTN---------
AtROD1            MMSKRH-LGTGFSLI---SKDSLVN----------
RcROD1            SKRKQAVV-AK-----ESSLFS-------------
Brassica          MMSKRHNLANGFSLI---SKDSLVN----------
Brachypodium      SKNVDSGENR--------CCSSCQK-ALFSQKLTS
Gossypum          SRRKSAVA-SGTMRLG-------------------
Glycin            SKRNAALS-TTHR-----AQFDCVNNVDIAKKINK
Hordeum           SKNVDSGDSR--------CCSSCQK-ALVSEKLTS
Medicago          DSKLKIAK-NSVNGHA--SKHDLVA----------
Oryza             GKNTVDGGAAVAPG---SRCCSCHK-ALLSQ----
Picea             SKRSTVQKDLVFEPLIG------------------
Populus           WKQRKPIA-NTVTVKDGRRLF--------------
Sorghum           AKNGD-GKNAPEKH---CRSCQCHK-ALLSH----
Helianthus        QRCGG------------------------------
Zea               AKNGD-SSNAPEKQ---CRSCQCHK-ALLSH----
```

Figure S1: **:** Comparison of deduced amino acid sequences of LuPDCT1, LuPDCT2, RcROD1, AtROD1 and PDCT-like proteins from other plant species. Sequences were aligned using Clastal Omega and identical amino acids are indicated with stars. the protein sequences were deduced from GenBank accession numbers: *Ricinus communis* (RcROD1), XM_002517597; *Arabidopsis thaliana* (AtROD1), At3g15820; *Brachypodium distachyon*, XP_003563650; *Glycin max*, XP_003528315; *Hordeum vulgare*, BAK03357; *Medicago truncatula*, XP_003604371; *Picea sitchensis*, ABK25679; *Populus trichocarpa*, XP_002327418; *Sorghum bicolor*, XP_002437259; *Zea maze*, NP_001145186; *Oryza sativa*, NP_001058029. The *Brassica napus*, *Gossypum spp*., *Helianthus annuus* sequences were obtained from the computational biology and functional genomic website (http://compbio.dfci.harvard.edu/compbio) with these gene identities; *Bn*, TC171107; *Gs*, TC240631; *Ha*, TC54879.

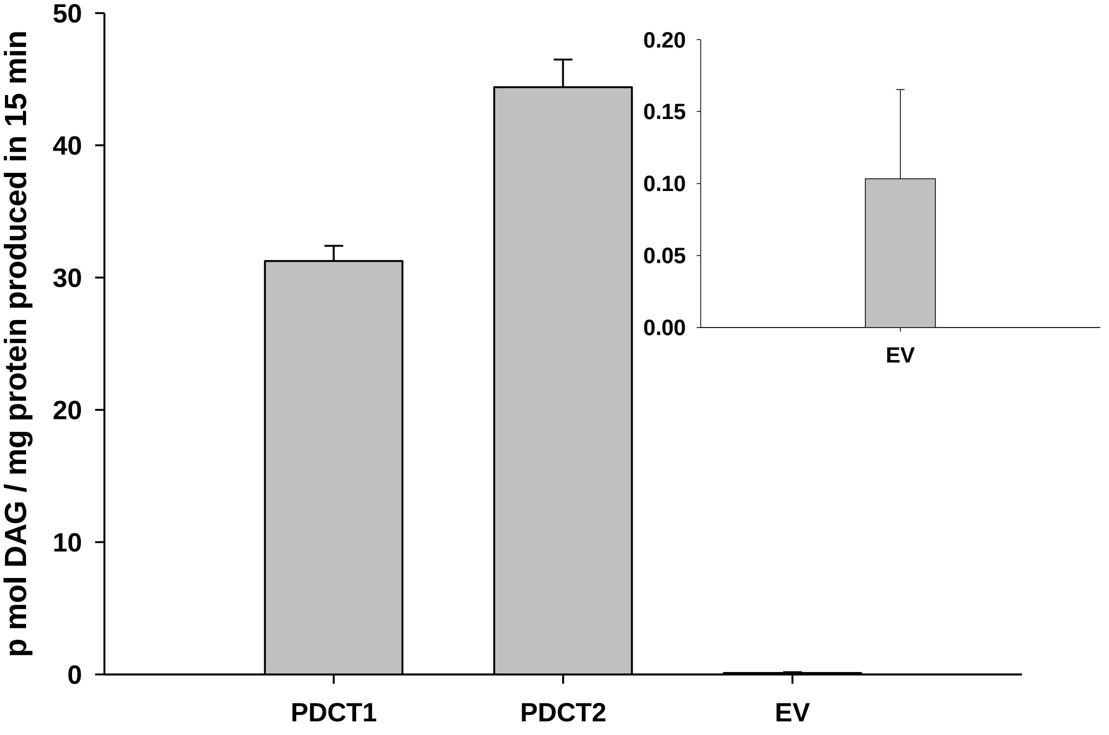

Figure S2: Production of radio-labeled diacylglycerol (DAG) catalyzed by LuPDCT1 or LuPDCT2 after 15 min incubation. Data shown represent means ± SE, with sample numbers of *n*=3

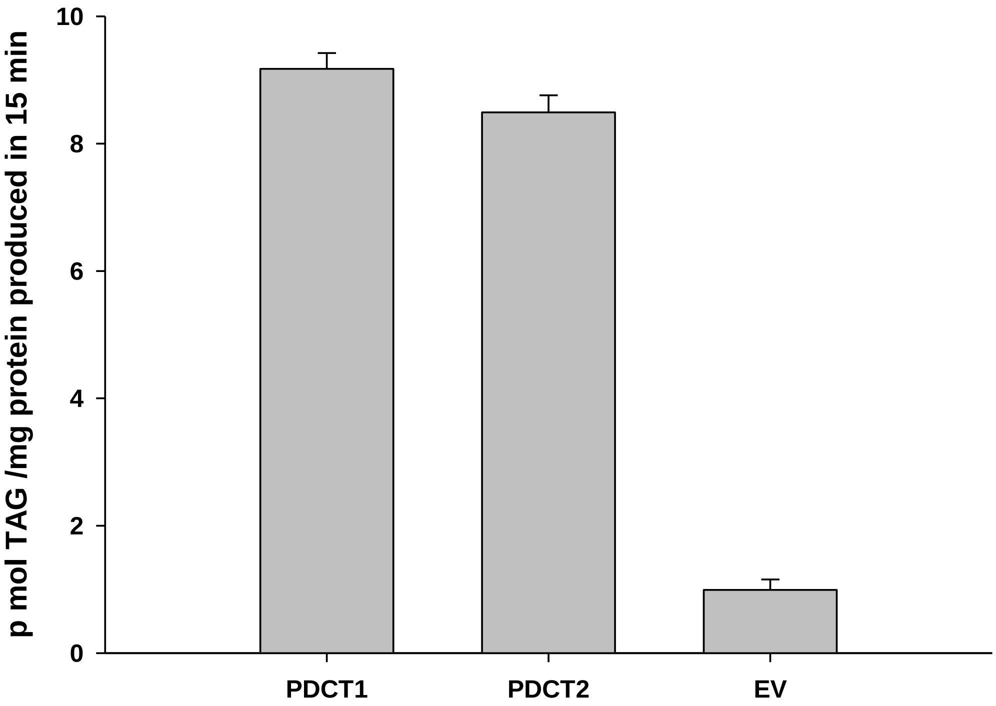

Figure S3: Production of radio-labeled triacylglycerol (TAG) during the assay of PDCT in the direction of radio-labeled diacylglycerol (DAG) formation. The incubation time was 15 min. Data shown represent means ± SE, with sample numbers of n=3

S Table 1: Proportions of C18:1, C18:2 and C18:3 in non-transformed wild-type (WT) and *rod1* mutant Arabidopsis seeds, as well as T_2 *rod1* seeds bearing empty vector, *LuPDCT1* (P1) or *LuPDCT2* expression cassettes. Results represent the average (±SE) of triplicate measurements of 10 independent transgenic lines for *PDCT* genes and five independent transgenic lines for empty vector (EV) transformed lines. For WT and *rod1* mutant lines, n=3.

Line	Fatty acid composition (mol %)		
	C18:1	C18:2	C18:3
WT	16.48±0.66	28.11±0.33	16.33±0.11
rod1	32.87±0.65	14.81±0.47	13.99±0.48
rod1+EV	37.74±0.32<br>(36.75-38.53)[a]	13.21±0.31<br>(12.26-13.45)	12.36±0.49<br>(10.67-13.37)
rod1+PDCT1	20.62±1.00<br>(15.40-24.29)	25.79±0.28<br>(24.55-27.44)	15.29±0.75<br>(12.86-19.03)
rod1+PDCT2	21.38±0.52<br>(18.20-22.77)	26.04±0.46<br>(23.62-28.02)	15.00±0.60<br>(12.76-17.80)

[a] Percent molar range among independent transformants.

S Table 2: Fatty acid composition of transgenic *A. thaliana* T_2 seeds expressing *LuPDCT1* and *LuPDCT2*. Results represent the average (±SE) of triplicate measurements of 10 independent transgenic lines for *PDCT* genes and five independent transgenic lines for empty vector (EV) transformed lines. For wild type (WT) lines n=5.

Construct	Fatty acid composition (mol %)									
	C16:0	C18:0	C18:1	C18:2	C18:3	C20:0	C20:1	C20:2	C22:1	18:2+18:3
PDCT1	9.77±0.13 (9.14-10.27)[a]	3.77±0.05 (3.50-3.92)	12.08±0.28 (9.99-12.99)	29.28±0.12 (28.66-29.95)	20.87±0.36 (19.52-22.83)	1.76±0.02 (1.67-1.90)	18.03±0.23 (17.21-19.44)	2.83±0.04 (2.67-3.12)	1.61±0.05 (1.40-1.93)	50.15±0.27 (49.08-51.49)
PDCT2	9.61±0.18 (8.82-10.55)	3.57±0.10 (3.10-4.24)	12.04±0.30 (9.73-13.28)	29.11±0.34 (27.78-31.64)	22.47±0.40 (20.09-24.24)	1.68±0.09 (1.24-2.35)	17.18±0.53 (13.51-19.35)	2.81±0.10 (2.30-3.20)	1.52±0.12 (1.02-2.29)	51.59±0.61 (49.35-55.84)
EV	8.71±0.15 (8.44-9.28)	4.71±0.27 (4.00-5.68)	17.90±0.21 (17.17-18.39)	26.43±0.36 (25.82-27.81)	16.67±0.28 (15.67-17.36)	2.75±0.18 (2.28-3.16)	18.83±0.45 (17.29-19.04)	2.36±0.06 (2.31-2.57)	1.82±0.03 (1.74-1.92)	43.10±0.57 (41.76-43.10)
WT	8.94±0.13 (8.54-9.16)	4.24±0.18 (3.76-4.65)	18.77±0.19 (18.26-19.31)	26.90±0.36 (25.69-27.79)	16.31±0.21 (15.86-16.98)	2.25±0.13 (1.81-2.60)	18.35±0.09 (18.09-18.64)	2.34±0.09 (20.9-2.54)	1.78±0.17 (1.46-2.38)	43.21±0.53 (41.56-43.62)

[a] Percent molar range among independent transformants.

S Table 3: Primers and probes used for qRT-PCR assay.

Gene	Forward primer	Reverse primer	Probe
PDCT1	TGCGATGAATGCGGTTAGG	CAACAGGGCACCTCCCAATA	AGCATTGGTTGCCTGTC
PDCT2	ACACCATTGATTTGGCCGCC	GGCGGTCTTGGTTTGGAGG	CTGCTCTTTGACTCACTTG
GAPDH	GACATCGTCTCCAACGCTAGCT	CATTGATAACCTTGGCCAAAG	ACCACTAACTGCCTTGC
Ubi2	CGGACACCATCGACAATGT	TCCGGCGGGATTCCTT	AAGGCCAAGATCCAG
